# Capacity of Health Facilities to Manage Hypertension in Mukono and Buikwe Districts in Uganda: Challenges and Recommendations

**DOI:** 10.1371/journal.pone.0142312

**Published:** 2015-11-11

**Authors:** Geofrey Musinguzi, Hilde Bastiaens, Rhoda K. Wanyenze, Aggrey Mukose, Jean-Pierre Van geertruyden, Fred Nuwaha

**Affiliations:** 1 Department of Disease Control and Environmental Health, School of Public Health, College of Health Sciences, Makerere University, Kampala, Uganda; 2 Primary and Interdisciplinary Care, University of Antwerp, Antwerp, Belgium; 3 Department of Epidemiology and Biostatistics, School of Public Health, College of Health Sciences, Makerere University, Kampala, Uganda; 4 International Health, Department of Epidemiology and Biostatistics, University of Antwerp, Antwerp, Belgium; University at Buffalo, UNITED STATES

## Abstract

**Background:**

The burden of chronic diseases is increasing in both low- and middle-income countries. However, healthcare systems in low-income countries are inadequately equipped to deal with the growing disease burden, which requires chronic care for patients. The aim of this study was to assess the capacity of health facilities to manage hypertension in two districts in Uganda.

**Methods:**

In a cross-sectional study conducted between June and October 2012, we surveyed 126 health facilities (6 hospitals, 4 Health Center IV (HCIV), 23 Health Center III (HCIII), 41 Health Center II (HCII) and 52 private clinics/dispensaries) in Mukono and Buikwe districts in Uganda. We assessed records, conducted structured interviews with heads of facilities, and administered questionnaires to 271 health workers. The study assessed service provision for hypertension, availability of supplies such as medicines, guidelines and equipment, in-service training for hypertension, knowledge of hypertension management, challenges and recommendations.

**Results:**

Of the 126 health facilities, 92.9% reported managing (diagnosing/treating) patients with hypertension, and most (80.2%) were run by non-medical doctors or non-physician health workers (NPHW). Less than half (46%) of the facilities had guidelines for managing hypertension. A 10^th^ of the facilities lacked functioning blood pressure devices and 28% did not have stethoscopes. No facilities ever calibrated their BP devices except one. About a half of the facilities had anti-hypertensive medicines in stock; mainly thiazide diuretics (46%), beta blockers (56%) and calcium channel blockers (48.4%). Alpha blockers, mixed alpha & beta blockers and angiotensin II receptor antagonists were only stocked by private clinics/dispensaries. Most HCIIs lacked anti-hypertensive medicines, including the first line thiazide diuretics. Significant knowledge gaps in classification of patients as hypertensive were noted among respondents. All health workers (except 5, 1.9%) indicated that they needed additional training in hypertension management. Several provider and patient related challenges were also observed in this study.

**Conclusions:**

Health facilities in this setting are inadequately equipped to provide services for management of hypertension. Diagnostic equipment, anti-hypertensive drugs and personnel present great challenges. To address the increasing burden of hypertension and other chronic diseases, measures are needed to substantially strengthen the healthcare facilities, including training of personnel in management of hypertension and other chronic diseases, and improving diagnostic and treatment supplies.

## Introduction

The epidemiological transition in global health from infectious to chronic non-communicable diseases (NCDs), especially, systemic hypertension, cardiovascular disease (CVD) and diabetes poses a significant threat to the health of those affected and the health systems at large[1]. More than three quarters (79%) of all deaths due to chronic diseases are occurring in developing countries and it is estimated that more than 60% of the burden of chronic diseases will occur in developing countries by 2020 [2,3]. Moreover, infectious diseases continue to disproportionately affect these countries with most deaths occurring due to malaria, tuberculosis, HIV and other infectious diseases [4]. Countries experiencing a double burden of disease must ration their meager resources to address the eminent dual epidemic of chronic and non-chronic diseases[5,6]. Current literature shows that acute infectious communicable diseases still contribute the major disease burden in sub-Saharan Africa including Uganda with malaria, acute respiratory infections and HIV/AIDS among the top 10 causes of illness and deaths[7]. However, with ageing populations, rising incomes, and increased exposure to behavioral risk factors contributing to new patterns of illness, disability and premature death due to NCDs, a greater policy attention to NCDs is warranted. A recent survey in Uganda shows that more than one in five patients have uncontrolled hypertension [8]. Implementing essential interventions for NCDs at lower level health facilities has the potential to prevent complications due to NCDs through early detection and treatment of people at high risk[9]. But, managing NCDs including hypertension is a daunting task in many facilities in low and middle income countries[10]. Socioeconomic barriers and inequalities in access to treatment, suboptimal staffing of health-care facilities and limited capacity to conduct investigations are some of the factors affecting management of NCDs [11–13]. As a strategy to curb NCDs, the World Health Organisation (WHO) and the International Society of Hypertension (ISH) advocates for blood pressure (BP) lowering and treatment of high-risk populations[14]. For least resources settings which are usually staffed by non-medical doctors or non-physicians health workers (NPHW), the WHO recommends a series of services which include BP measurement, history taking to elicit antecedents of heart attack, angina and stroke, counselling on behaviour modification (physical activity, diet, cessation of tobacco smoking), measurement of body mass index (BMI), administration of 1^st^ line thiazide and prompt referral[14]. The Uganda clinical guidelines (UCG) stipulate a range of recommendations for classification, diagnosis and management of hypertension. According to these guidelines, patients are classified as hypertensive if they present with a persistently high resting blood pressure (SBP ≥ 140 mmHg or DBP ≥ 90 mmHg or both). Most of these are asymptomatic and are often discovered on routine examination. The UCG recommendations for management of hypertension include: 1) advice on life style modification (diet, cessation of alcohol drinking and tobacco smoking, and weight reduction) for mild hypertension in the first three months or otherwise administer a thiazide diuretic when all lifestyle advice fails; 2) treat moderate or severe hypertension with a thiazide diuretic plus an AC inhibitor, Or angiotensin II receptor antagonist, Or beta blockers. The guidelines also provide additional recommendations on management of patients with complications such as stroke, heart failure, kidney disease, post myocardial infarction, coronary artery disease and diabetes.

The most commonly reported NCD through the health management information system in Uganda is hypertension. Moreover, hypertension offers a useful entry point for management of NCDs[15]. Appropriate management of hypertension would require trained providers with the necessary equipment and medicines. There is little documentation of the status of healthcare facilities in sub-Saharan Africa and their level of preparedness to offer appropriate management for hypertension[16,17]. In this study, we report the capacity of health facilities to manage hypertension in two districts in Uganda.

### The Uganda national health system

The Uganda National Health System (NHS) is made up of the public and the private sectors. The public sector includes all government health facilities under the Ministry of Health (MoH) and other line ministries. The private health delivery system consists of Private Health Providers (PHPs), Private Not for Profit (PNFPs) providers and the Traditional and Complimentary Medicine Practitioners (TCMPs). The PNFPs are integrated in the Ministry of Health service delivery framework. However, many PHPs run private facilities which are not aligned to the Uganda Ministry of Health service delivery system. Nevertheless, relevant legislation exists that provides for licensing and regulation of health professionals who engage in private practice[18].

### The district healthcare delivery framework

The district health system operates under a decentralized frame work and follows a referral system from a lower facility to a higher facility. The public facilities provide free services but in many cases units don't have the essential drugs, meaning that patients have to buy them from pharmacies or drug sellers. Details of **the Health care delivery and referral system in Uganda are as illustrated in S1 Fig.** At the village level (HCI) are village health teams (VHT) whose roles are among others to advice, refer patients to facilities and distribute essential medicines. Next is a HCII facility at a parish level. The HCII runs an out-patient clinic, treating common diseases and offering antenatal care. The third level, HCIII is found at a sub-county and run a general outpatient clinic and a maternity ward. The HCIV and general hospitals serve a county or a parliamentary constituency. They provide preventive medicine, health promotion, curative maternity, in-patient health services, surgery, blood transfusion, laboratory and medical imaging services. They also provide in-service training, consultation and operational research in support of the community-based health care programs.

## Methods

### Setting

From June to October 2012, we conducted a cross sectional survey of health facilities in two districts (Mukono and Buikwe) in Uganda. The two districts combined had 145 health facilities (6 hospitals, 4 HCIVs, 27 HCIIIs, 57 HCIIs and 52 private clinics/dispensaries). Mukono had 90 health facilities (1 general hospital, 4 Health Centre (HC) IVs, 14 HC IIIs, 26 HC IIs and 46 registered private clinics/dispensaries). Buikwe had 55 health facilities (5 general hospitals, 13 HC IIIs, 31 HCIIs and 6 registered private clinics/dispensaries). In this study, we defined private clinics/dispensaries as–those private healthcare facilities which were run by PHPs and not integrated into the Ministry of Health service delivery frame work but were registered to provide curative services (e.g. consultations, disease diagnosis, drug dispensing and treatment) by their respective districts.

### Study design

**Fig 1:**
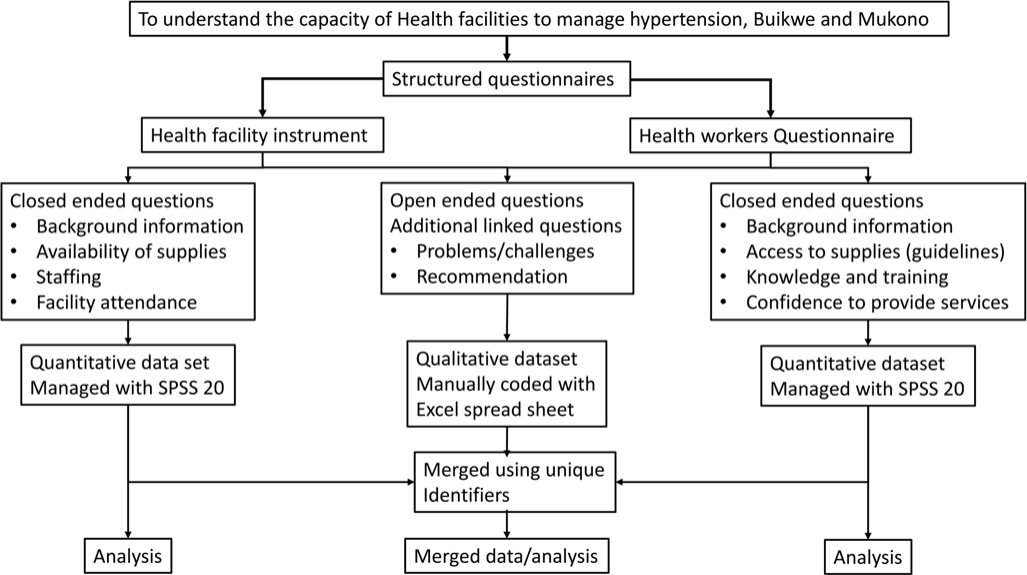
Framework illustrating the study design and data structure.

This was a cross sectional study that employed concurrent mixed method of data collection [19]. Concurrent collection of both structured and unstructured data was necessary to enable collection of linked data from the same respondents. Topic-specific structured questions were followed by linked open-ended questions, when necessary. In addition, open-ended responses were useful in triangulation of some of the quantitative findings at analysis level. Fig 1 illustrates the conceptual framework of the mixed methods approach and the relevant data collected.

### Selection of health facilities

Of the 145 registered health facilities in the two districts, 126 (86.9%) were included in this study. These were 6 hospitals, 4 HCIVs, 23 HCIIIs, 41 HCIIs and 52 private clinics/dispensaries. For purposes of generating sufficient samples for health facility level comparisons, all hospitals, HCIV and registered private clinics/dispensaries in the study districts were enrolled since these were few. For HCIIIs and HCIIs we randomly selected; 23 and 44 facilities respectively. These numbers were deemed sufficient for analysis of correlations/comparisons. The sampling process is illustrated in the supporting information, S2 Fig.

### Selection of health workers

Providers who were working in the outpatient unit of the facilities were eligible for the health workers questionnaire. These included doctors, clinical officers, nurses/midwives and nursing assistants. Study respondents per level of facility and within facility levels were selected proportionate to size.

### Data collection

The data for this survey were generated using a set of two modified and pretested tools (a health facility instrument and a health workers questionnaire)[17,20]. The data were collected by a team of trained research assistants under the supervision of two of the lead investigators (GM and FN). Key variables collected and how they were defined are as illustrated in S1 Table.

### The health facility instrument

The instrument had both structured questions and a checklist component for data extraction from facility records. The health facility data collection instrument gathered information on background of the health facility such as type of facility and ownership, involvement of the facility in diagnosing/treating patients with hypertension, total outpatient and hypertension attendances, availability of hypertension guidelines, anti-hypertensive medicines and stock out, diagnostic equipment (such as BP machines, cuffs, and stethoscopes) and human resources. We administered closed questions to health facility in-charges or designees, conducted observations and reviewed records to extract relevant data. In addition, we used a semi-structured interview schema to give participants the opportunity to elaborate on problems, challenges and suggestions for improvement.

### The Health workers Questionnaire

The health workers’ questionnaire was used to assess background characteristics of respondents, knowledge on hypertension management (including levels at which respondents would classify patients as hypertensive, life style modification advice), frequency of providing hypertension related services, having attended any in-service courses on hypertension management, whether respondents needed additional training in hypertension management, confidence in providing the different services, and guidelines. Besides the structured closed questions, open ended questions were included to allow for elaboration on problems, challenges and recommendations. Doctors, clinical officers, nurses, midwives and nursing assistants responded to the health workers questionnaire.

### Statistical analysis

**Fig 2:**
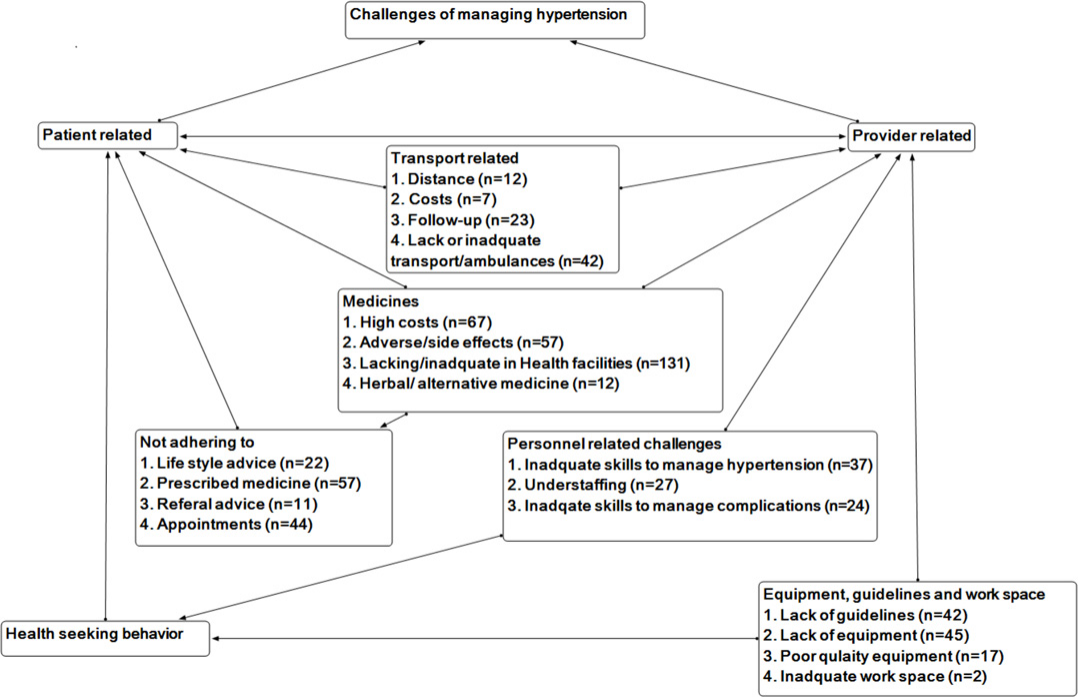
Graphical presenation of the challenges of managing hypertension in Mukon and Buikwe District in Uganda.

Quantitative data were analysed using IBM SPSS statistics version 20 to generate descriptive statistics, which were summarised using proportions. To assess whether certain facilities were very well stocked in all dimensions compared to others in terms of vital supplies such as guidelines, equipment and medicines and whether the vital supplies were related to the volume of hypertension patients, we assessed correlation between these items. To assess whether facilities with broken equipment were more likely to be those with working equipment or not, we assessed correlation between functioning BP devices and non-functioning devices by facility level. Comparisons of categorical outcomes by facility level were generated using contingency tables. The relationship between continuous and dichotomous variables were conducted using simple linear regression. The following groups of parameters were compared: (1) availability of guidelines versus number of hypertensive patients; (2) drug stock out versus number of hypertensive patients; (3) Availability of BP devices versus number of hypertensive patients; (4) BP device (available and functioning versus non-functioning); (6) availability of guidelines vs. BP devices vs. drugs. Because of in-applicability of some questions to some facilities, the denominator varied in certain instances for each analysis conducted. Chi-square and the Fisher exact test were used to test relationships between two categorical variables. Unpaired t-test was used to measure the relationship between continuous and dichotomous variables. Alpha was set at 0.05 for significance testing. Open-ended questions were analyzed and quantitized using a coding framework with tally sheets in Microsoft Excel. The quantitized qualitative data was merged with the quantitative data for comparative analysis, Fig 2. Graphical presentations were generated using Atlas ti version 7.

### Ethics statement

This study received approval from the Makerere University School of Public Health Higher Degree Research and Ethics Committee and the Uganda National Council of Science and Technology. Written informed consent was obtained from all health workers who participated in the study.

## Results

### Background characteristics

The basic characteristics of the studied health facilities and the study respondents are summarised in Table 1. Fifty-one (40.5%) of the facilities were government owned, 52 (41.3%) were private for profit (PFP) and the rest were private not for profit (PNFPs) (either mission or NGO affiliated). Of the 126 health facilities, 92.9% reported diagnosing and/or treating patients with hypertension and most (98.3%) facilities managed hypertension in a general outpatient facility. The average patient attendance (all cases) at the health facilities in July 2012 were 2562 in hospitals, 1792 in HCIV, 1108 in HCIII, 474 in HCII, and 165 in Private clinics/dispensaries and the mean number of hypertension attendance per facility in the same month were 39 in hospitals, 19 in HCIV, 13 in HCIII, 1 in HCII and 4 in Private clinics/dispensaries. Majority of the health workers were females (68.3%), Christians (82.4%) and their mean age was 35.4 (SD±10.8). They comprised of doctors (n = 21), clinical officers (n = 60), nurses/midwives (n = 136), nursing assistants (n = 51), others (n = 2) and (n = 1) had missing data.

**Table 1:** Distribution of studied baseline characteristics by level of facility in Mukono and Buikwe districts in Uganda.

Characteristics	Total N (%)	Hospital = 6; N (%)	HCIV = 4; N (%)	HCIII = 23; N (%)	HCII = 41; N (%)	Clinic/dispensary = 52; N (%)
**Studied health worker participants; n = 271**						
**Mean age (SD) = 35.4 (10.8))**						
**Sex**						
Female n(%)	185 (68.3)	38 (67.9)	13 (76.5)	42 (72.4)	48 (77.4)	44 (56.4)
**Cadre (N = 268)**						
Doctor	21 (7.8)	7 (12.5)	1 (5.6)	0 (0.0)	0 (0.0)	13 (16.9)
Clin. Officer	60 (22.5)	11 (19.6)	8 (44.4)	18 (32.1)	4 (6.6)	19 (24.7)
Nurse/Midwif	136(50.7)	37 (66.1)	6 (33.3)	30 (53.6)	28 (45.9)	35 (45.5)
N/Assistant	51 (19.0)	1 (1.8)	3 (16.7)	8 (14.3)	29 (47.5)	10 (13.0)
**Studied health facilities (N = 126)**						
**Health facility (Ownership)**						
Government	51 (40.5)	1 (16.7)	3 (75.0)	20 (87.0)	27 (65.9)	-
Private	52 (41.3)	-	-	-	-	52 (100)
NGO/Mission	23 (18.2)	5 (66.7)	1 (25.0)	3 (13.0)	14 (34.1)	-
**Health facility diagnose or/and treat hypertension**						
Yes, n (%)	117 (92.9)	6 (100)	4 (100)	23 (100)	40 (97.6)	44 (84.6)
**Health facility average number of outpatient attendance (July 2012)**						
All cases Mean (SD)	660.1 (844.2)	2562.3 (2040.9)	1791.6 (1447.1)	1108.1 (457.4)	474.4 (224.2)	165.3 (277.1)
Hypertensive Mean (SD)	7.0 (12.1)	39.3 (24.4)	19.5 (15.3)	13.0 (12.2)	1.15 (3.4)	4.04 (4.5)

### Guidelines, equipment and personnel

Table 2 reveals that less than a half (46%) of the health facilities had hypertension guidelines (exclusively UCG) with significant differences observed across health facility levels (p<0.001). Very few (18%) private clinics/dispensaries had guidelines. More hospitals (83.3%) and HCIIIs (73.9%) had guidelines. More than 10%, of the facilities (HCIII [13%], HCII [14.6%] and Private clinics/dispensaries [7.7%]) lacked functioning BP devices. Facilities with BP devices had mainly aneroid (67.7%) and mercury (19.1%) sphygmomanometers and a few digital devices (13.1%) were mostly found in private/dispensaries (79.3%), (p<0.001). Except one health facility, all never calibrated their sphygmomanometers. There were notable discrepancies between functioning BP measuring devices and the cuffs. Seventeen percent of the health facilities lacked standard cuffs, and cuffs for obese patients and children were extremely scarce. Although the difference was not statistically significant (P = 0.73), a significant proportion of health facilities (28%); mostly private clinics/dispensaries and HCIIs lacked stethoscopes. Of all the facilities, only 19.8% (25/126) had physicians (medical doctors) with statistically significant difference in distribution across facilities (P<0.001). Most (66.6%, 18/27) of the physicians were in private clinics, 29.6% (8/27) were in hospitals and 3.7% (1/27) at HCIV.

**Table 2:** Availability of guidelines and functioning equipment for hypertension diagnosis and management.

Variable	Total	Hospital = 6	HCIV = 4	HCIII = 23	HCII = 41	Clinic/dis	P-Value
	N (%)	N (%)	N (%)	N (%)	N (%)	pensary = 52; N (%)	
**Hypertension guidelines at the health facility (N = 124)**							
Yes	57 (46)	5 (83.3)	2 (50)	17 (73.9)	24 (58.5)	9 (18)	<0.001
**Availability of blood pressure measuring devices at health facility (N = 126)**							
Yes	113 (89.7)	6 (100)	4 (100)	20 (87)	35 (85.4)	48 (92.3)	0.63
**Number and type of blood pressure measuring device in (N = 113) health facilities**							
Digital	29 (13.2)	0 (0)	2 (11.8)	1 (2.9)	3 (6.3)	23 (30.3)	<0.001
Aneroid	149 (67.7)	33 (73.3)	10 (58.8)	27 (79.4)	40 (83.3)	39 (51.3)	0.12
Mercury	42 (19.1)	12 (26.7)	5 (29.4)	6 (17.6)	5 (10.4)	14 (18.4)	0.002
**Calibration of the BP apparatus (N = 108)**							
Never	107 (99.1)	6 (100)	4 (100)	23 (0)	41(0)	51 (98.1)	
**Cuffs for measuring blood pressure (N = 108)**							
Standard cuff	159 (98.8)	35 (100)	5 (100)	29 (100)	33 (100)	57 (96.6)	<0.001
Small cuff	1 (0.6)	0 (0)	0 (0)	0 (0)	0 (0)	1 (1.7)	
Large cuff	1 (0.6)	0 (0)	0 (0)	0 (0)	0 (0)	1 (1.7)	
**Availability of stethescopes (N = 126)**							
Yes	91 (72)	6 (100)	4 (100)	21 (91.3)	33 (80.5)	27 (51.9)	0.73
**Mean number of stethescopes per level of health facility**							
N (Mean)	169 (1.3)	32 (5.3)	13 (3.3)	44 (1.9)	47 (1.1)	33 (0.6)	
**Availability of medical doctors (physicians) at health facilities (N = 126)**							
Yes	25(19.8)	6 (100)	1 (25)	0 (0)	0 (0)	18 (34.6)	<0.001
**Number of doctors (overall and per health facility level)**							
Doctors	27	8 (29.6)	1 (3.7)	0 (0)	0 (0)	18 (66.7)	
**Drug stock out**							
Yes	29 (34.1)	3 (50)	1 (25)	6 (26)	2 (33.3)	17 (36.9)	0.80

P- Value is significant at P < 0.05

### Medicines

Table 3 shows availability of anti-hypertensive medicines at different health facilities in Mukono and Buikwe districts in Uganda. Significant differences between stocked drugs and health facility levels were observed for diuretics, beta blockers, calcium channel blockers, ACE inhibitors, angiotensin receptor II agonists and alpha 2 agonists (All p<0.001). Proportions of available anti-hypertensive medicines (at all levels) were; 42.9% for thiazide diuretics, 59% beta blockers, 1.6% alpha blockers, 5.6% mixed alpha and beta blockers, 48.4% calcium channel blockers, 22.2% angiotensin converting enzyme (ACE) inhibitors, 15.9% angiotensin II receptor antagonists, and 16.6% alpha-2 agonists. Stratification by level of facility shows that all hospitals and HCIVs (except one) had thiazide diuretics, beta blockers, calcium channel blockers, and ACEs; but none stocked alpha blockers, and mixed alpha & beta blockers. Very few (16.6%) health facilities stocked alpha-2 agonist, and only one HCIV stocked angiotensin II receptor antagonists. Meanwhile, more than 90% of HCIIs did not stock anti-hypertensive medicines. On the other hand, significant proportions of private clinics/dispensaries stocked beta blockers (97.6%) and calcium channel blockers (80.8%), (P<0.001). The data also reveal that a wide range of antihypertensive medicine were available in quite a number of private clinics/dispensaries including mixed alpha and beta blockers (13.7%), ACEs (36.5%), angiotensin II receptors antagonists (34.6%) and alpha-2 agonist (34.6%). Regarding drug stock outs, 50% of the hospitals and 26% of HCIIIs reported stock outs in the three months preceding the study although the difference in reported stock out was not statistically significant (p = 0.80). Drawing from Table 2 and Table 3, facilities in this setting are inadequately prepared to manage hypertension. In fact the joint probability of any one facilities (hospitals, HCIVs, HCIIIs, and HCIIs) having all relevant variables (medicines, calibrated equipment, personnel, etc) occurring simultaneously was zero given that none of them had their BP calibrated at least once in the past 6 months preceding the study.

**Table 3:** Availability of different anti-hypertensive medicines by health facility level.

Class of	Total	Hospital	HCIV = 4	HCIII = 23	HCII = 41	Clinic/dis	P-Value
medicine*	N (%)	= 6	N (%)	N (%)	N (%)	pensary =	
		N (%)				52; N (%)	
**Diuretics**							
Yes	54 (42.9)	6 (100)	4 (100)	17 (73.9)	4 (9.8)	23 (44.2)	<0.001
**Beta blockers**							
Yes	75 (59.5)	6 (100)	3 (75)	22 (95.6)	4 (9.8)	40 (97.6)	<0.001
**Alpha blockers**							
Yes	2 (1.6)	0 (0)	0 (0)	0 (0)	0 (0)	2 (3.8)	0.576
**Mixed alpha and beta Blockers**							
Yes	7 (5.6)	0(0)	0 (0)	0 (0)	0 (0)	7 (13.5)	0.032
**Calcium channel blockers**							
Yes	61 (48.4)	6 (100)	4 (100)	3 (13)	6 (14.6)	42 (80.8)	<0.001
**Angiotensin converting enzyme (ACE) inhibitors**							
Yes	28 (22.2)	5 (75)	3 (75)	1 (4.3)	0 (0)	19 (36.5)	<0.001
**Angiotensin II receptor antagonists**							
Yes	20 (15.9)	0 (0)	1 (25)	0 (0)	1 (2.4)	18 (34.6)	<0.001
**Alpha-2 agonists**							
Yes	21 (16.6)	2 (33.3)	1 (25)	0 (0)	0 (0)	18 (34.6)	<0.001

*Reported drugs

Diuretics–Hydrochlorothiazide and chlorothiazide; Bendroflumethiazide, Spironolactone, Amiloride

Beta Blockers–Atenelol, Propranolol

Alpha blockers–Indormine, Phenoxybenzamine

Mixed Alpha and Beta Blockers–Carvedilol, Labetalol

Calcium Channel Blockers–Amlodipine, Nifedipine, Nimodipine

Angiotensin Converting Enzyme (ACE) Inhibitors–Captopril, Enalapril

Angiotensin II Receptor antagonists–Losartan

Alpha-2 agonists–Methyldopa

P- Value is significant at P < 0.05

### Knowledge, training and confidence in hypertension management

To asses existing gaps in knowledge and abilities of health workers to offer hypertension related services, health workers were asked a range of questions about access to guidelines and their knowledge of levels at which to classify patients as hypertensive, BP levels at which they prescribe anti-hypertensive medicines, their need for additional training in management of hypertension and their confidence in provision of hypertension related services. Providers were asked if they had ever seen guidelines for management of patients with hypertesnion and 58.3%, (154/264) had never seen the guidelines. Forty seven percent (127/269) diagnosed hypertension at systolic blood pressure (SBP) of 140mmHg and 57.6% (155/269) diagnosed hypertension at diastolic blood pressure (DSP) of 90mmHg. The remaining health workers either diagnosed patients at higher or lower values. For example, a significant proportion, 37.9% (102/269) of health workers diagnosed patients as hypertensive at SBP<140mmHg and 14.1% (38/269) diagnosed patients as hypertensive at DBP<90mmHg. Composite analysis of reported systolic-diastolic blood pressure cut offs revealed that 9% (25/269) diagnosed patients as hypertensive at systolic/diastolic pressure <140mmHg and <90mmHg. With regard to anti-hypertensive treatment, about 26% (68/266) prescribed anti-hypertensive drugs for patients with SBP <140mmHg and 10.9% (29/269) prescribed drugs for patients with diastolic blood pressure <90mmHg. On a likert scale, 76.3% of health workers strongly agreed or agreed that most patients with hypertension are asymptomatic, 12.2% neither agreed nor disagreed whereas 11.5% disagreed or strongly disagreed. Regarding additional training in the management of hypertension in the two years preceding the study, only 18.1% (49/217) had attended a course encompassing hypertension management. Most providers said that they were confident in handling various aspects of hypertension management such as measuring blood pressure, 97.4% (261/268), advising on; salt intake 93.3% (252/270), diet 94.9% (256/270), exercise 95.1% (257/270), alcohol consumption 93.7% (252/269), smoking 90.3% (242/268), and prescribing drugs 86.8% (231/266). On the other hand, several health workers were not confident in calculating BMI 37.5% (101/269), and managing a patient with stroke 51.8% (139/268). In contrast to the high confidence, almost all respondents, 98.1% (261/266) indicated a need for additional training in hypertension care.

### Correlation between sub groups of selected outcome variables by Health facility level

Table 4 shows a statistically significant positive correlation between availability of guidelines and the volume of patients at the hospital level (R = 0.957, P = 0.003). On the other hand, there was no significant correlation between having guidelines and the volume of hypertensive patients at the rest of the health facility levels. No significant correlation was observed between drug stock out and the volume of patients except at the private clinics/dispensaries where a statistically significant negative correlation was observed (R = -0.341, p<0.022). Similarly, there was no significant correlation between functioning BP devices with volume of hypertensive patients at all facility levels except at the private clinic/dispensary where a statistically significant positive correlation was observed (R = 0.572, p<0.001). Likewise, there was no significant correlation between functioning BP devices and non-functioning devices except at the private clinics/dispensary where a statistically significant positive correlation was observed (R = 0.592, p<0.001). At all levels, no significant correlation was observed between guidelines vs. drug stocks vs. BP devices.

**Table 4:** Statistical analysis comparing correlation coefficients (R) of sub groups of outcome variables with Health facility level.

	Guidelines	Stock out	Function BP device	Functioning BP
	vs.	Vs	vs.	vs
Health facility type	Patient number	Patient number	Patient number	Non functioning
HOSPITAL	**0.957** ^*^	-0.479	0.512	0.566
HCIV	-0.643	0.197	0.502	0.801
HCIII	-0.1.14	0.196	0.031	-0.327
HCII	0.085	-0.592	-0.122	0.194
Private clinic/Dispensary	0.035	-0.341*	0.572**	0.592**

**. Correlation is significant at the 0.01 level (2-tailed).

*. Correlation is significant at the 0.05 level (2-tailed).

### Challenges in managing patients with hypertension

With an open-ended question, health workers were asked to mention the challenges they face in managing hypertension. Fig 2 shows a conceptual presentation of the stated challenges, which we have categorized into patient related and provider related challenges. On the patient side, providers reported that patients do not adhere to treatment advice. Several of them defaulted medicines, scheduled appointments, and lifestyle and referral advice. Attributable causes of defaults were reported to include: high costs of drugs, transport especially for patients from rural areas, drug stock out at public facilities, adverse effects and resorting to traditional medicine. In addition, providers reported that many patients also delayed to seek care–they reported to facilities only when they had life threatening triggers. On the provider side, mainly medicines, equipment and personnel challenges were reported. Respondents reported that public facilities were never adequately equipped with medicines. Consequently, patients were provided with prescription to buy the anti-hypertensive medicines but some never did so. Inadequate medicines and stock out were reported at all health facility levels with HCIIIs and HCIIs reporting acute understocking. Understaffing and inadequate skills to manage hypertension and related complications were also reported at the hospitals as well as the lower level facilities.

### Suggested recommendation by health workers to improve hypertension management

**Fig 3:**
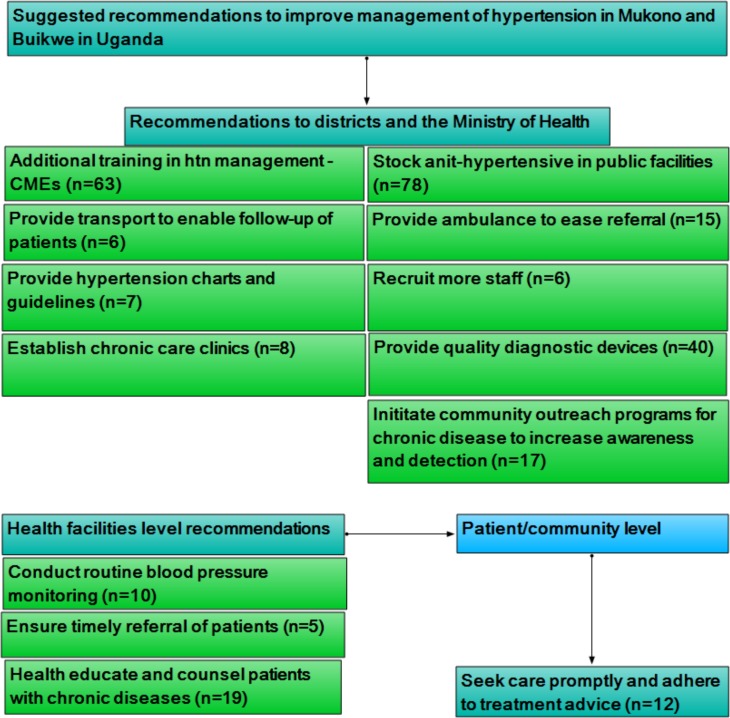
Suggested recommendations to improve hypertension management in Mukono and Buikwe in Uganda.

Following the stated challenges, we asked health workers to suggest recommendations for improvement. Fig 3 shows suggested recommendations which we have classified as policy, health facility and community/patient level recommendations. Policy recommendations from the data were related to provision of continuing medical education in hypertension, supplies and medicines, and initiating community interventions to increase awareness and detection of hypertension and other chronic diseases. The need to train health workers in hypertension management was reported at all health facility levels including the private clinics/dispensaries. Equipping public health facilities with anti-hypertensive medicines was mentioned by 78 (53.8% (78/145) health workers in public facilities. Twenty (25.6%) of these were from hospitals, 12 (15.4%) from HCIVs, 13 (16.7%) from HCIIIs and 33 (42.3%) from HCIIs. For the diagnosis of hypertension, the respondents recommended that facilities should be provided with high quality diagnostic equipment, citing that some of their equipment were of poor quality and provided inaccurate measurements. Regarding personnel, they recommended additional recruitment and attraction of higher skilled personnel to lower level health facilities to aid in managing hypertension and other chronic diseases. At health facility level, especially lower levels such as HCII, HCIII and HCIV, they recommended that patients should be routinely monitored for blood pressure and promptly referred. To address patient related challenges such as non-adherence and poor health seeking behaviours, health providers suggested that motivational counselling, patient education on chronic diseases, establishment of chronic care units and community outreach programs should be instituted. But for outreach and improvement in referral system, they noted the need to provide transport for outreach services and ambulances to health facilities. With regards to hypertension guidelines, health workers said that they needed to be provided with guidelines, hypertension management charts and information, education and communication (IEC) materials to enhance their performance. At the community or patient level, they encourage patients to seek care promptly and also adhere to treatment advice to prevent complications and mortalities.

## Discussion

In this study, which assessed the capacity of health facilities to manage hypertension in two districts in Uganda, 92.9% of the 126 health facilities were diagnosing and/or treating hypertension. Most of the facilities were run by NPHW. Most HCIIs didn’t stock anti-hypertensive medicine. About a half of all the facilities stocked thiazide diuretics, beta blockers and calcium channel blockers with more hospitals, HCIV and private clinics/dispensaries reporting stock availability compared to HCIII and HCII. A wide range of anti-hypertensive drugs were common in several private clinics/ dispensaries including angiotensin receptor antagonists, alpha II agonists and mixed alpha and beta blockers. None of the facilities (except one) calibrated their BP devices. The commonest BP devices were aneroid and mercury sphygmomanometer. More than a half of the facilities had no access to hypertension guidelines. This probably explains why a significant proportion of health workers could not correctly state blood pressure cut offs. Moreover, an overwhelming proportion expressed the need for additional training in hypertension management. Challenges for managing hypertension were further related to patients’ failure to adhere to treatment advice, late reporting and health system weaknesses such as understaffing, inadequate medicines and equipment. These findings highlight gaps in provision of care for hypertension and other chronic diseases in this low income context. They provide additional evidence to existing literature in sub-Saharan Africa[16,17,21–23] about health systems preparedness to manage hypertension and the increasing burden of chronic disease.

When physicians are scarce, in the face of dramatic increase in chronic disease and other institutional and social economic challenges, a paradigm shift in the health work force may be the only option available to address the burden of chronic diseases[22]. According to Abegunde and colleagues, non-physician health workers are compelled to care for patients with chronic diseases although they may not be trained to recognize them or make robust, timely and life-saving treatment decisions [11]. In our study, most facilities were run by non-physicians who were also responsible for managing hypertension. Unfortunately, substantial proportions lacked guidelines and didn’t seem to be adequately knowledgeable to manage hypertension.

As previously reported in other settings, medicines, and diagnostic equipment for management of hypertension were inadequate [16,17] and negatively impacted on health seeking behavior and treatment out comes for patients[24]. In Tanzania, authors reported that basic supplies were absent at many health facilities, especially the lower level health facilities[16]. In a study by White, patients diagnosed with hypertension were given medicines for one to two weeks and told to return for follow-up. Although shorter schedules were a monitoring strategy, White also noted that inadequate drugs and monitoring devices partly contributed to the treatment decisions. Providers used it as a strategy to minimize stock out. Unfortunately, after reviews of out-patient registers in government health units, she discovered that the names of those given medicines for a short time did not usually reappear within the month[25]. Moreover, lack of medicine and diagnostic equipment at public health facilities meant that patient seek services from private providers at a price which often excluded poor patients from accessing them. This scenario creates some concerns about equitable access to hypertension services. Based on our community survey in the districts of Mukono and Buikwe, more than 60% of the study population earned less than US$200 every month[26]. Moreover, more than two thirds of the population resided in rural Mukono and Buikwe where private clinics/dispensaries were scarce. These rural populations instead largely depended on the HCIIIs and HCIIs, which were under equipped with diagnostic devices, medicines and personnel.

In order to cope with the higher burden of hypertension and other chronic disease, substantially strengthening of primary health facilities will be warranted.[27] Such strengthening would focus on major aspects of chronic care delivery including task shifting, training of providers, timely and adequate provision of medicines and equipment, and prompt referral.

### Limitations

This study did not ascertain diagnostic accuracy and appropriate management of hypertension. However, the study highlights glaring capacity gaps that need to be addressed to enhance prevention and management of hypertension in a low-income context. This being a cross sectional study, trends in stock out were not captured. Moreover, inferring cause-effect relationship was not feasible but the findings provide useful data for hypertension policy improvement and programing.

## Conclusions

Health facilities in this setting are inadequately equipped to provide services for hypertension management. Essential ingredients for management of hypertension including, diagnostic equipment, anti-hypertensive medicines and personnel present great challenges. To address the increasing burden of hypertension and other chronic diseases, measures are needed to substantially strengthen the healthcare facilities including training of personnel in hypertension management and improving diagnostic and treatment supplies.

## Supporting Information

S1 Fig Illustrates the Health care delivery and referral system in Uganda. (PDF)

S2 Fig Flow diagram showing the sampling criteria of Health facilities. (PDF)

S1 Table Shows details of key variables collected and how they were defined. (PDF)
