# Supplementary figures and images for: Capacity of Health Facilities to Manage Hypertension in Mukono and Buikwe Districts in Uganda: Challenges and Recommendations
Source: PLoS One. 2015 Nov 11;10(11):e0142312. doi: 10.1371/journal.pone.0142312 (PMC4641641; doi:10.1371/journal.pone.0142312)

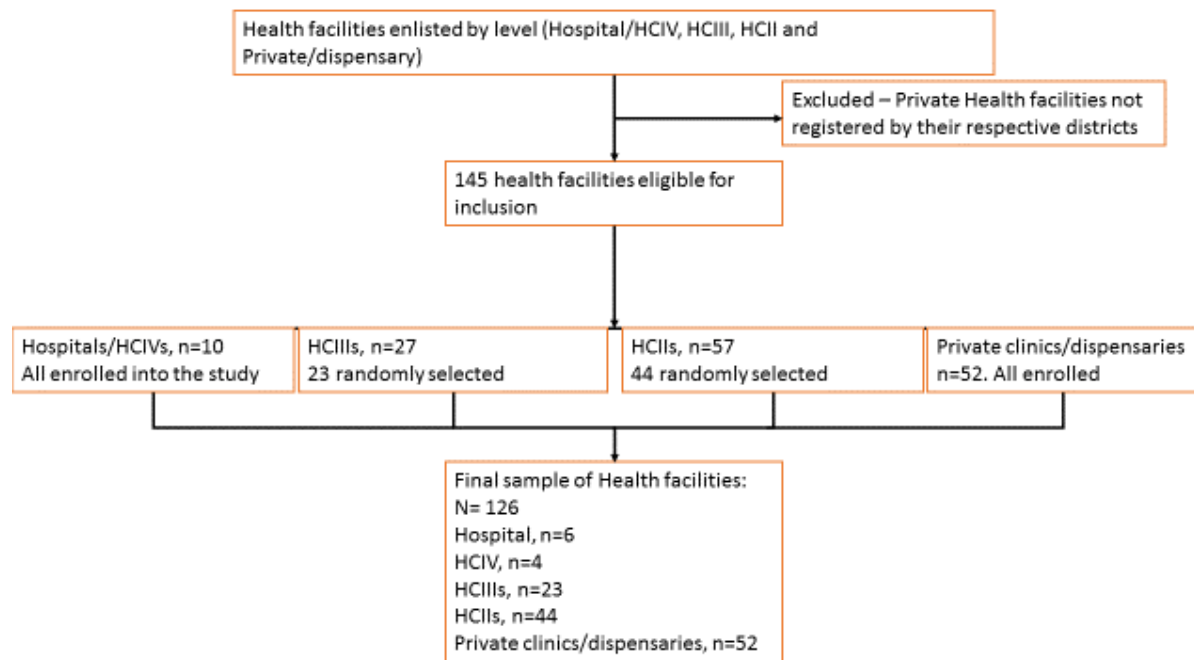

**S2 Figure: Flow diagram showing the sampling criteria of Health facilities**

Supplement: S2 Fig — (PDF) [file pone.0142312.s002.pdf]
