# Supplementary material for: Capacity of Health Facilities to Manage Hypertension in Mukono and Buikwe Districts in Uganda: Challenges and Recommendations
Source: PLoS One. 2015 Nov 11;10(11):e0142312. doi: 10.1371/journal.pone.0142312 (PMC4641641; doi:10.1371/journal.pone.0142312)
Supplement: S1 Table — (PDF) [file pone.0142312.s003.pdf]

S1 Table: shows details of key variables collected and how they were defined

Variables	Variable definition/measurement
Availability of hypertension guidelines	Guidelines observed at the health facility
Access to guidelines	Respondents were asked whether they had had guidelines at their facilities for management of hypertension
Management of hypertension	A facility was classified as managing hypertension if it was involved in one or more of the following: (1) diagnosing patients for hypertension; (2) treating patients for hypertension
Total attendance	Total number of outpatient visits in the month of July
Hypertension attendance	Number of outpatient visits related to hypertension in the month of July
Availability of anti-hypertensive	Any antihypertensive (Thiazide diuretics, Beta blockers, Alpha blockers, Mixed alpha and beta blockers, Calcium channel blockers, Angiotensin converting enzymes, Angiotensin II receptor antagonist and Alpha-2 agonists) observed at the health facility
History of drug stock out	History of drug stock out was enlisted by asking facility in charges whether they had run out of anti-hypertensive in the last 3 months
Availability of diagnostic equipment (blood pressure device [Digital, aneroid, mercury], Stethoscope, cuff [standard, for children and for obese], weighing scale)	The facility had diagnostic equipment (Blood pressure apparatus, manual or digital, stethoscopes, cuff, and weighing) during the time of the survey
Staff training on hypertension	Any staff member having received training in the diagnosis and management of hypertension within the past 2 years
Calibration of BP devices	Calibration was measured by asking facility in charges – (How often the BP machines are calibrated)
Classification of a patient as hypertensive	Health workers were asked – (At what blood pressure would you classify a patient as hypertensive?<br>a) Systolic=<br>b) Diastolic =
Confidence to provide a service (BP measurement, life style advice such as a advice on salt, managing patient with stroke)	Providers were defined as confident if they reported very confident or confident on a likert scale of 1-5, where by one was very confident and 5 was strongly not confident.
